# Adaptation and Validation of the Bullying and Cyberbullying Scale for Adolescents in Bangla

**DOI:** 10.1002/brb3.71093

**Published:** 2025-11-25

**Authors:** Humayra Shahjahan, Ahsan Aziz Sarkar, Shelina Fatema Binte Shahid, Nahid Mahjabin Morshed

**Affiliations:** ^1^ Child and Adolescent Psychiatry LifeSpring Consultancy Limited Dhaka Bangladesh; ^2^ Dhaka Division Bangladesh Ministry of Health and Family Welfare Directorate General of Family Planning Dhaka Bangladesh; ^3^ Clinical Psychology, Department of Psychiatry Bangabandhu Sheikh Mujib Medical University Dhaka Bangladesh; ^4^ Child and Adolescent Psychiatry, Department of Psychiatry Bangabandhu Sheikh Mujib Medical University Dhaka Bangladesh

**Keywords:** Bangla, BCS‐A, bullying, cyberbullying, validation

## Abstract

**Introduction:**

Adolescent bullying is a pressing global public health and educational issue, including in Bangladesh. The lack of valid, reliable assessment tools in clinical and school settings impedes the identification of victims and individuals involved in bullying and cyberbullying. This study aimed to adapt and validate the Bullying and Cyberbullying Scale for Adolescents (BCS‐A) in Bangla.

**Methods:**

This cross‐sectional study followed a standard adaptation protocol to translate the BCS‐A into Bangla. The study included 210 randomly selected adolescents (12–17 years) from three Bangla‐medium schools in Dhaka. Participants completed the Bangla‐adapted BCS‐A, a sociodemographic questionnaire, and the Strengths and Difficulties Questionnaire (SDQ).

**Results:**

The mean score of the victimization subscale was 9.60 (SD: ±9.1) and the perpetration subscale was 5.15 (SD: ±6.55). The Cronbach's alpha values were 0.821 and 0.808 for the victimization and perpetration subscales, respectively, indicating robust reliability. For the majority of items, the item‐total correlation was above 0.30. Confirmatory factor analysis indicated a four‐factor structure for each of the subscales, aligning with previous studies. Victims of bullying showed a positive correlation with internalizing problems on the SDQ, whereas perpetrators of bullying showed a positive correlation with externalizing problems, suggesting good convergent and divergent validity. A total victimization score of ≥ 5 and a total perpetration score of ≥ 4 demonstrated the ability to accurately identify victims and perpetrators, respectively, with over 80% sensitivity and specificity.

**Conclusion:**

The BCS‐A Bangla has demonstrated strong reliability and validity, making it a useful tool for screening bullying and cyberbullying behaviors among adolescents.

## Graphical abstract

This cross‐sectional validation study adapted and assessed the Bangla BCS‐A among adolescents, demonstrating strong reliability and validity. As the first validated tool in Bangladesh for comprehensive screening of bullying and cyberbullying in a single setting, it supports early identification of victims and perpetrators in clinical and educational contexts.

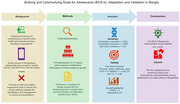

## 1 Introduction

Adolescence is a critical period of growth; however, this phase can also be marked by struggles such as bullying and cyberbullying, which can have long‐lasting negative effects on mental health and development (Thomas et al. 2016; Wolke and Lereya 2015). Bullying involves repeated behaviors intended to cause harm, often occurring in situations of power imbalance (Gladden et al. 2014). The rise of digital platforms has further complicated this issue, leading to the emergence of cyberbullying (Thomas et al. 2015). Globally, bullying victimization affects approximately 24.3% of adolescents, while cyberbullying impacts 11.1% (Li et al. 2024). Like many countries in Asia (Man et al. 2022), Bangladesh is also experiencing emerging concerns related to bullying and cyberbullying. Over the past three decades, rapid economic development has been accompanied by a substantial rise in secondary school enrollment from about 20% three decades ago to roughly 70% now (UNICEF Bangladesh 2020). At the same time, mobile phone and internet use have become widespread among adolescents, with nearly 90% reporting mobile phone use and 60% having internet access (Yousuf and Yousuf 2023). Parallel to these trends, reported incidents of both bullying and cyberbullying have increased severalfold, with studies indicating that 35% of adolescents report experiencing bullying (Ahmed et al. 2021) and 31.9% report being victims of cyberbullying (Mallik and Radwan 2020). These behaviors are associated with serious consequences, including physical and psychological harm as well as poor academic performance (Kowalski and Limber 2013). Effective tools are needed to assess both traditional bullying and cyberbullying, especially among adolescents (Gladden et al. 2014; Thomas et al. 2015).

The Bullying and Cyberbullying Scale for Adolescents (BCS‐A), developed by researchers at Queensland University, provides a comprehensive tool to measure both bullying and cyberbullying victimization and perpetration (Thomas et al. 2019). The BCS‐A has been successfully adapted and validated in countries such as Turkey (Özbey and Başdaş 2020), Iran (Ammar 2021), and Indonesia (Santhoso et al. 2023), which share cultural similarities with Bangladesh. While validated instruments like the Bangla version of the Olweus Bully/Victim Questionnaire‐Revised (OBVQ‐R) exist (Mahmood et al. 2023), no comprehensive tool in Bangladesh currently assesses both traditional bullying and cyberbullying within a single framework. This gap underscores the need for a culturally relevant, dual‐measure instrument to accurately capture the full scope of bullying behaviors among Bangladeshi adolescents. Such a tool will assist clinicians in identifying and assessing bullying behaviors and support school counselors and relevant bodies in formulating targeted and effective anti‐bullying strategies. This, in turn, will help mitigate these issues and promote safer environments (Modecki et al. 2014). This study aimed to validate the Bangla version of the BCS‐A in Bangladesh, facilitating early detection, risk assessment, and intervention strategies to address bullying and cyberbullying among adolescents.

## 2 Methods

### 2.1 Study Design

This cross‐sectional validation study was carried out in 2023–2024 at the Psychiatry Department of Bangladesh Medical University (BMU), Dhaka. Permission for adaptation and validation of BCS‐A in Bangla was obtained from the author of the BCS‐A tool and the copyright holders. Ethical approval was secured from the Ethics Committee of BMU, Dhaka (BSMMU/2023/9371). The study population consisted of students aged 12–17 years from Grades 6 to 12 of either sex, selected through a mixed sampling method where schools were purposively chosen and students were randomly selected from each grade.

### 2.2 Adaptation Process

The adaptation of the BCS‐A ratio scoring version into Bangla followed the standardized cross‐cultural adaptation protocol outlined by Beaton et al. (2000). Two bilingual professionals, one child psychiatrist and one linguist, independently translated the original English version into Bangla. The initial translations were reconciled during a consensus meeting involving both translators and a third reviewer, resulting in a single forward translated version. This version was then back translated into English by an independent translator who was unfamiliar with the original scale to ensure conceptual equivalence. Any discrepancies between the original and back translated versions were discussed and resolved through iterative group review until consensus was reached.

To ensure cultural appropriateness, an expert panel consisting of a psychiatrist, clinical psychologist, school counselor, and community development worker reviewed each item for conceptual, experiential, and idiomatic equivalence. Cultural relevance was further assessed through cognitive debriefing interviews with 20 adolescents from diverse socioeconomic backgrounds. These participants provided feedback on item clarity, relevance, and understanding. Final modifications were made based on both expert feedback and adolescent input, producing the finalized Bangla adapted version of the BCS‐A.

Both ratio and ordinal scoring versions of the BCS‐A were translated into Bangla. Cognitive debriefing interviews with adolescent participants revealed that the ratio scale format, which records the frequency of bullying and cyberbullying behaviors over the past 3 months using discrete count categories (never, once, twice, thrice, four, or more times), was easier to understand and respond to than the ordinal scale format. The ordinal scale employs broader frequency descriptors such as “did not happen to me,” “once or twice,” “every few weeks,” “about once a week,” and “several times a week or more,” which participants found less intuitive. Consequently, and following expert committee consultation emphasizing clarity and respondent comprehension, the ratio scoring version was selected for validation.

### 2.3 Sample and Data Collection

Data were collected from three secondary and higher secondary Bangla‐medium schools in Dhaka, Bangladesh: one boys' school, one girls' school, and one coeducational school. The inclusion criteria comprised students enrolled in Grades 6–12, aged 12–17 years, who had used the internet for at least the past 3 months, ensuring relevance to the Bangladeshi schooling system. Students with cognitive impairment due to underlying medical or psychiatric conditions were excluded from the study. The sample size was determined using a participant‐to‐item ratio of 6:1, with an adjustment for a 5% margin of missing data, resulting in a total of 210 participants. This approach aligns with recommended guidelines for validation studies (Anthoine et al. 2014). This sample size was also consistent with guidelines for confirmatory factor analysis (CFA), which recommend a minimum of 200 participants or a participant‐to‐item ratio ranging from 5:1 to 10:1, depending on model complexity and factor loadings (Kyriazos 2018). Schools were selected purposively, while students were chosen through simple random sampling. A total of 70 students were enrolled from each school, with 10 students randomly selected from each grade.

The students and their parents were informed about the study's purpose, ethical considerations, and their right to withdraw. Written informed consent was obtained from the parents, and informed assent was obtained from the students.

### 2.4 Measures

This study utilized a semi‐structured questionnaire to collect sociodemographic data, along with the Bangla‐adapted version of the BCS‐A and the Bangla version of the Strengths and Difficulties Questionnaire (SDQ).

The BCS‐A, developed by Thomas et al. (2019), comprises two parallel 13‐item scales: one measuring victimization and the other assessing bullying, each further divided into four subscales (physical, verbal, relational, and cyberbullying). The scale was originally constructed by integrating items from various validated instruments, including the OBVQ‐R (Olweus 1996), with a reference period of the previous 3 months. Physical bullying items address behaviors such as hitting, pushing, or damaging belongings, while verbal items focus on name‐calling and hurtful language. Relational bullying involves exclusion from groups and spreading rumors, and cyberbullying encompasses online harassment, including mean messages, hurtful posts, and digital exclusion. The original version demonstrated good concurrent validity with the OBVQ‐R and the SDQ, as well as strong test‐retest reliability and predictive validity for internalizing and externalizing problems. The BCS‐A has also shown strong psychometric properties in different countries, including good reliability with Cronbach's alpha up to 0.93 (Özbey and Başdaş 2020; Ammar 2021), and excellent model fit indices such as the Comparative Fit Index (CFI) and the Tucker–Lewis Index (TLI) above 0.95 with the root mean square error of approximation (RMSEA) below 0.08 (Santhoso et al. 2023). These results support the scale's reliability and validity across various cultural settings. The scale employs a 5‐point scale incorporating both ratio and ordinal measurements with two distinct scoring systems. In this study, a ratio scale was used, where responses were recorded as the frequency of occurrence (e.g., 0, 1, 2, 3, 4, 5, etc.).

Adolescents' internalizing and externalizing problems were evaluated using the Bangla self‐report version of the SDQ (Mullick and Goodman 2001). The internalizing problems score was calculated by combining the emotional problems and peer problems subscales, while the externalizing problems score was calculated by combining the conduct problems and hyperactivity subscales. Greater psychological difficulties are indicated by higher ratings.

### 2.5 Statistical Analysis

Data analysis was conducted using IBM SPSS Statistics 27. The Shapiro–Wilk test of normality indicated significant departures from normality for all items of the BCS‐A (all *p* < 0.001), supporting the use of nonparametric analytical approaches. The reliability of the scale was assessed through Cronbach's alpha coefficient to measure internal consistency for the victimization and perpetration subscales, as well as for each of the four domains (physical, verbal, relational, and cyberbullying). Item‐total correlations were also computed. CFA was conducted using the lavaan package in R (version 4.5.1) with the diagonally weighted least squares (DWLS) estimator to validate the original four‐factor structure of both the victimization and perpetration subscales. The hypothesized model specified four latent factors corresponding to physical, verbal, relational, and cyberbullying domains. Model fit was assessed using the chi‐square to degrees of freedom ratio (*χ* ^2^/df), CFI, TLI, and RMSEA. Acceptable model fit was defined by CFI and TLI values of 0.90 or higher and RMSEA values below 0.08, with a *χ* ^2^/df ratio less than 3 indicating good fit. The convergent and divergent validity was evaluated by correlating the domain scores of the BCS‐A with internalizing and externalizing problem scores on the SDQ.

Optimal cutoff points were determined for two populations: (1) victims and perpetrators and (2) victims with internalizing problems and perpetrators with externalizing problems. To identify victims and perpetrators, adolescents were classified based on a score of three or more in any domain or total subscale of the BCS‐A, categorizing them accordingly into the respective domains and subscales. To enhance the clinical applicability of the BCS‐A Bangla, we defined victims with internalizing problems as individuals scoring ≥ 3 on any victimization subscale domain or the total victimization subscale of the BCS‐A, in addition to scoring ≥ 7 on the emotional problems domain or ≥ 6 on the peer problems domain of the SDQ. Similarly, perpetrators with externalizing problems were identified as those scoring ≥ 3 on any perpetration subscale domain or the total perpetration subscale of the BCS‐A, along with a score of ≥ 5 on the conduct problems domain or ≥ 7 on the hyperactivity domain of the SDQ. This classification aligns with previous recommendations that define frequent victimization and bullying as occurring three or more times within the past month (Solberg and Olweus 2003). The clinical relevance of this categorization is further supported by the observed association of victimization with internalizing problems (Hawker and Boulton 2000) and bullying perpetration with externalizing problems (Kelly et al. 2015). To determine the cutoff points, receiver operating characteristic (ROC) curve analysis was conducted. Sensitivity and specificity were calculated for optimal cutoff points of each domain and subscale of the BCS‐A to accurately identify victims and perpetrators.

### 2.6 AI Use Disclosure

The authors used OpenAI's ChatGPT (version GPT‐5, accessed in 2025) to assist in improving language clarity and grammar in portions of the manuscript draft. The authors reviewed and edited all output to ensure accuracy and originality. No AI‐generated content was used in data analysis or interpretation.

## 3 Results

Data from 202 adolescents (mean age = 14.46 years, SD: ±1.74) were analyzed after excluding 8 cases with missing data. The male‐to‐female ratio was approximately 1:1, with students from girls' schools (35.1%), boys' schools (31.7%), and coeducational schools (33.2%) constituted near equally. The majority of adolescents lived in nuclear families (86.6%) and identified as Muslim (93.1%).

### 3.1 Reliability

Table 1 presents the item characteristics of the Bangla version of the BCS‐A, including Cronbach's alpha values for individual domains and subscales, mean scores with standard deviations, corrected item‐total correlations, and the impact of item deletion on internal consistency. The Cronbach's alpha values for individual domains ranged from 0.393 to 0.691 for the victimization subscale domains and from 0.482 to 0.681 for the perpetration subscale domains, while the overall subscale reliability was 0.821 for victimization and 0.808 for perpetration subscale, respectively. The corrected item‐total correlation values were greater than 0.3 for all items, except for VP3, VR1, VR2, PP3, and PC2. However, removing the weakly correlated items did not result in a significant change in the Cronbach's alpha values for the respective subscales. The mean score of the victimization subscale was 9.60 (SD: ±9.1) and the perpetration subscale was 5.15 (SD: ±6.55). The mean scores for individual items varied, with higher scores observed for verbal victimization (VV2: 1.56 ± 1.85) and verbal perpetration (PV1: 1.19 ± 1.93), suggesting that these behaviors were more frequently reported.

**TABLE 1:** Item characteristics of BCS‐A Bangla.

Item	Cronbach's alpha (individual domain)	Cronbach's alpha (subscale)	Mean ± SD	Corrected item‐total correlation	Cronbach's alpha if item deleted
**Victimization subscale**					
VP1	0.583	0.821 (*ω* = 0.810)	0.92 ± 1.37	0.449	0.809
VP2			0.64 ± 1.16	0.454	0.805
VP3			0.52 ± 1.17	0.243	0.817
VP4			0.73 ± 1.28	0.327	0.816
VV1	0.594		1.44 ± 1.69	0.423	0.808
VV2			1.56 ± 1.85	0.423	0.799
VR1	0.393		0.44 ± 0.91	0.263	0.820
VR2			0.95 ± 1.40	0.263	0.794
VC1	0.691		0.79 ± 1.38	0.524	0.802
VC2			0.28 ± 0.79	0.309	0.819
VC3			0.22 ± 0.72	0.397	0.817
VC4			0.51 ± 1.47	0.483	0.807
VC5			0.60 ± 1.20	0.580	0.796
**Perpetration subscale**					
PP1	0.623	0.808 (*ω* = 0.700)	0.55 ± 1.11	0.425	0.805
PP2			0.37 ± 1.07	0.437	0.794
PP3			0.15 ± 0.53	0.264	0.801
PP4			0.16 ± 0.57	0.493	0.800
PV1	0.681		1.19 ± 1.93	0.523	0.782
PV2			0.78 ± 1.46	0.523	0.796
PR1	0.482		0.28 ± 1.59	0.318	0.792
PR2			0.19 ± 0.63	0.318	0.801
PC1	0.595		0.73 ± 1.32	0.422	0.778
PC2			0.13 ± 0.48	0.263	0.802
PC3			0.17 ± 0.65	0.449	0.797
PC4			0.18 ± 0.65	0.454	0.795
PC5			0.14 ± 0.62	0.325	0.799

Abbreviations: PC, perpetration cyber; PP, perpetration physical; PR, perpetration relational; PV, perpetration verbal; VC, victimization cyber; VP, victimization physical; VR, victimization relational; VV, victimization verbal; *ω*, McDonald's omega.

### 3.2 Validity

The Bangla version of the BCS‐A demonstrated adequate face validity, with both expert reviewers and lay participants confirming the tool's clarity and appropriateness for assessing bullying and cyberbullying behaviors. Content validity was rigorously established during the initial development of the original instrument. The four‐factor structure of Bangla BCS‐A subscales was confirmed using CFA. The CFA of the victimization subscale showed a *χ* ^2^ value of 89.57 (df = 59, *p* = 0.006), with a *χ* ^2^/df ratio of 1.52, an RMSEA of 0.046, a CFI of 0.965, and a TLI of 0.954, indicating a good model fit. Similarly, the perpetration subscale demonstrated a *χ* ^2^ value of 78.10 (df = 59, *p* = 0.049), a *χ* ^2^/df ratio of 1.32, an RMSEA of 0.051, a CFI of 0.965, and a TLI of 0.953, also reflecting an acceptable fit. These results suggest that the proposed four‐factor structure of the BCS‐A Bangla version aligns well with the data, supporting its validity. Table 2 shows unstandardized loading, standard error, and standardized loadings for each item of BCS‐A.

**TABLE 2:** Standardized factor loadings for victimization and perpetration subscales (four‐factor model).

	Victimization subscale			Perpetration subscale		
Domain	Unstd. loading	SE	Std. loading	Unstd. loading	SE	Std. loading
Physical	1.00^a^	—	0.57	1.00^a^	—	0.51
Physical	0.88	0.14	0.60	0.68	0.25	0.51
Physical	0.61	0.15	0.41	0.14	0.08	0.15
Physical	0.72	0.18	0.45	0.33	0.15	0.33
Verbal	1.00^a^	—	0.59	1.00^a^	—	0.86
Verbal	1.24	0.16	0.76	0.64	0.10	0.64
Relational	1.00^a^	—	0.38	1.00^a^	—	0.63
Relational	3.21	0.99	0.79	0.74	0.26	0.46
Cyber	1.00^a^	—	0.64	1.00^a^	—	0.76
Cyber	0.27	0.09	0.30	0.10	0.05	0.21
Cyber	0.29	0.09	0.36	0.29	0.10	0.44
Cyber	0.57	0.10	0.56	0.34	0.11	0.52
Cyber	1.06	0.15	0.79	0.16	0.07	0.27

*Note*: All loadings are statistically significant (*p* < 0.05). Standardized loadings represent the completely standardized solution.

Abbreviations: SE, standard error; Std., standardized; Unstd., unstandardized.

^a^ Indicates reference items fixed to 1.0 for model identification.

Table 3 presents the convergent and divergent validity of the BCS‐A Bangla version by correlating its domain scores with SDQ internalizing and externalizing problem scores. Victimization due to physical and verbal bullying showed significant positive correlations with internalizing problems (*r* = 0.258, *p* < 0.001; *r* = 0.266, *p* < 0.001), while relational and cyber victimization showed no significant associations. Conversely, perpetration through physical and verbal bullying significantly correlated with externalizing problems (*r* = 0.258, *p* < 0.001; *r* = 0.266, *p* < 0.001), with relational and cyberbullying perpetration also showing weaker but significant correlations.

**TABLE 3:** Convergent and divergent validity of BCS‐A Bangla assessed by correlating domain scores with the SDQ internalizing and externalizing problem scores.

	SDQ internalizing problem		SDQ externalizing problem	
	Correlation coef.	*p*	Correlation coef.	*p*
**Victimization subscale**				
Physical	0.258	< 0.001	0.050	0.480
Verbal	0.266	< 0.001	0.015	0.834
Relational	0.040	0.496	0.057	0.420
Cyber	0.010	0.834	0.035	0.658
**Perpetration subscale**				
Physical	0.049	0.489	0.258	< 0.001
Verbal	0.015	0.834	0.266	< 0.001
Relational	0.036	0.630	0.171	0.010
Cyber	0.030	0.671	0.204	< 0.001

### 3.3 Cutoff Points

We measured the proportion of adolescents who were victimized and perpetrated by interpreting BCS‐A ratio scale scores. For victimization, verbal bullying was the most prevalent, with 44.6% experiencing it three or more times, followed by physical (33.7%), cyber (26.7%), and relational (23.3%) victimization. In terms of perpetration, verbal bullying was the most common, with 24.8% engaging in it three or more times, while physical (12.4%), cyber (16.8%), and relational (5.9%) perpetration were less frequent. The ROC curve analysis was conducted to determine the optimal cutoff points, assessing the trade‐off between sensitivity and specificity for each subscale. Table 4 presents the sensitivity and specificity of selected cutoff points for identifying victims and perpetrators using the BCS‐A Bangla. A total victimization score of ≥ 5 demonstrated the optimal balance of sensitivity and specificity for identifying victims, while a total perpetration score of ≥ 4 was found to be the most effective cutoff for detecting perpetrators.

**TABLE 4:** Optimal cutoff points for identifying victims and perpetrators on the BCS‐A.

Domain	AUC (95% CI)	*p* value	Cutoff point (total score)	Sensitivity (%)	Specificity (%)	PPV (%)
**Victimization**						
Physical	0.84 (0.79–0.89)	< 0.001	4	85.3	91.8	85.8
Verbal	0.89 (0.84–0.93)	< 0.001	3	97.8	92.9	92.6
Relational	0.72 (0.66–0.79)	< 0.001	3	91.5	96.2	86.8
Cyber	0.78 (0.72–0.85)	< 0.001	4	83.3	93.2	80.4
Total victimization	0.95 (0.93–0.98)	< 0.001	5	95.9	82.7	45.5
**Perpetration**						
Physical	0.70 (0.61–0.78)	< 0.001	3	88.5	92.6	62.7
Verbal	0.80 (0.73–0.87)	< 0.001	3	90	90.8	75.0
Relational	0.61 (0.52–0.69)	0.009	3	58.3	98.4	67.6
Cyber	0.75 (0.67–0.82)	< 0.001	3	82.4	94.6	74.3
Total perpetrator	0.88 (0.82–0.93)	< 0.001	4	85.9	80.0	58.9

*Note*: PPV calculation is based on the observed rates of victimization and perpetration in the study sample.

Abbreviation: PPV, positive predictive value.

Among the adolescents, 24 (11.9%) were identified as victims with internalizing problems, while 17 (8.4%) were classified as perpetrators with externalizing problems. Table 5 presents the optimal cutoff points for identifying these groups. A total victimization score of ≥ 26 (mean ≥ 2) demonstrated 100% sensitivity and 32.6% specificity in identifying victims with internalizing problems. Similarly, a total perpetration score of ≥ 26 (mean ≥ 2) identified perpetrators with externalizing problems with 100% sensitivity and 50% specificity.

**TABLE 5:** Optimal cutoff points for identifying victims with internalizing and perpetrators with externalizing problems on the BCS‐A.

Domain	AUC (95% CI)	*p* value	Cutoff point (mean score)	Sensitivity (%)	Specificity (%)	PPV (%)
**Victimization**						
Physical	0.69 (0.58–0.80)	0.002	≥ 3.5	58.3	69.1	18.9
Verbal	0.68 (0.59–0.77)	0.003	≥ 2.5	75.0	56.2	17.6
Relational	0.54 (0.41–0.67)	0.468	≥ 2.5	37.5	78.1	16.6
Cyber	0.61 (0.50–0.72)	0.073	≥ 2.5	62.5	68.0	19.5
Total victimization	0.72 (0.64–0.80)	< 0.001	≥ 2.0	100	32.6	19.9
**Perpetration**						
Physical	0.67 (0.53–0.81)	0.016	≥ 1.0	61.7	67.4	14.7
Verbal	0.77 (0.66–0.88)	< 0.001	≥ 2.5	82.4	75.5	24.9
Relational	0.68 (0.53–0.83)	0.012	≥ 1.0	52.9	82.6	22.1
Cyber	0.76 (0.63–0.88)	< 0.001	≥ 1.0	67.6	69.1	16.8
Total perpetrator	0.84 (0.78–0.91)	< 0.001	≥ 2.0	100	50.0	15.5

*Note*: PPV calculation is based on the observed rates of the victims with internalizing and perpetrators with externalizing problems in the study sample.

Abbreviation: PPV, positive predictive value.

## 4 Discussion

Given the lack of a validated tool in Bangla, the aim of this study was to culturally adapt and validate the BCS‐A for Bangla‐speaking populations, providing a reliable tool for assessing Bangladeshi adolescents. The adaptation procedure followed a rigorous protocol maintaining international standards of adaptation. We randomly recruited participants representing Bangladesh's demographic and cultural diversity in terms of age, gender distribution, and family structure (Bangladesh Bureau of Statistics 2022).

The Cronbach's alpha values for each subscale exceeded the acceptable threshold of 0.70, indicating good internal consistency of the scale (Tavakol and Dennick 2011). However, individual domain alpha values fell within the questionable to unacceptable range, which is expected for shorter scales or domains with fewer items. Comparisons with the Turkish adaptation of the BCS‐A (Özbey and Başdaş 2020) revealed similar findings, with acceptable overall reliability (*α* > 0.7) despite lower domain‐specific alpha values. In contrast, the Egyptian version demonstrated excellent overall reliability (*α* = 0.90) (Ammar 2021). The reliability of the Bangla version was further supported by item‐total correlations exceeding 0.30 for most items, consistent with psychometric standards (Nunnally and Bernstein 1994). Moreover, removing four of the five items with lower item‐total correlation coefficients would not improve the overall reliability but rather decrease the Cronbach's alpha value. This suggests that the scale's psychometric properties are comparable to those found in culturally similar contexts.

The CFA supported the original four‐factor structure across both victimization and perpetration subscales, confirming the scale's construct validity. These results are consistent with the original scale development and subsequent validations in Egypt and Turkey, indicating the BCS‐A's stable factor structure across diverse populations. Importantly, the convergent and divergent validity of the Bangla BCS‐A was established through correlations with the SDQ. These findings align with a meta‐analytic review of two decades of cross‐sectional studies, which reported that bullying victimization is linked to higher rates of internalizing problems (Hawker and Boulton 2000). Likewise, perpetrators or bullies are more likely to exhibit externalizing problems (Kelly et al. 2015).

In clinical and research settings, establishing practical cutoff scores is vital for identifying adolescents at risk. Although the BCS‐A does not prescribe cutoff points, our findings suggest that total victimization and perpetration scores of ≥ 5 for total victimization score and ≥ 4 for total perpetration score appeared to provide optimal chances in identifying victims and perpetrators, respectively. Furthermore, a total subscale score of ≥ 26 demonstrated promise in screening for bullying‐related internalizing and externalizing problems, paralleling recommendations from prior studies (Ammar 2021). The tool demonstrated high sensitivity, meaning it effectively identifies true cases (victims or perpetrators), but its lower specificity suggests a higher chance of false positives. Given the complexities of bullying research, careful interpretation of these findings is necessary, as they may overestimate the psychological impact associated with bullying‐related experiences. The findings suggest that the BCS‐A Bangla is most effective as a tool for identifying victims and perpetrators of bullying rather than for detecting individuals with bullying‐related psychiatric disorders. While the scale demonstrates strong psychometric properties in distinguishing those involved in bullying behaviors, its ability to accurately identify victims and perpetrators with psychiatric conditions may be limited. Therefore, the BCS‐A Bangla should be utilized primarily as a screening instrument for bullying involvement, with additional clinical assessments required to evaluate associated psychiatric comorbidities.

The current findings corroborate existing evidence regarding the BCS‐A's psychometric properties, demonstrating that the Bangla adaptation is suitable for identifying bullying behaviors in both clinical practice and research applications. Nevertheless, certain methodological constraints warrant consideration. The sample was restricted to urban adolescent populations, potentially affecting the instrument's generalizability to rural contexts. Differences in digital literacy and internet access across rural regions may affect how adolescents interpret or respond to cyberbullying‐related items. Tailored studies in rural areas could help assess the scale's applicability in those settings and identify potential modifications to account for contextual differences. While the sampling procedure was random at the participant level, the purposive selection of schools may limit the representativeness of the findings, especially across regions with different educational infrastructures. Broader randomization strategies in future studies could mitigate this issue. In addition, the use of self‐report measures for sensitive topics such as bullying may have introduced social desirability bias, with participants potentially underreporting involvement in bullying or overreporting socially acceptable behaviors. Future studies could incorporate multi‐informant approaches or indirect assessment techniques to reduce this bias. The cross‐sectional nature of the study precluded assessment of test‐retest reliability and predictive validity. Longitudinal studies are necessary to evaluate the scale's capacity to predict long‐term outcomes of bullying involvement. Furthermore, the lack of validated Bangla‐language comparison measures limited the examination of concurrent and criterion validity, presenting challenges for comprehensive validation. The optimal cutoff scores identified in this study are sample‐derived and await validation in an independent external cohort. While sensitivity analyses by sex and grade would be informative, our sample size did not provide sufficient statistical power for such subgroup comparisons; this remains an important avenue for future research with larger, stratified samples.

## 5 Conclusion

In summary, the Bangla version of the BCS‐A demonstrates strong psychometric properties, offering a culturally relevant and reliable tool for identifying adolescents involved in bullying and cyberbullying behaviors. While the scale is best used as a screening instrument rather than a diagnostic tool for psychological disorders, its utility in both research and clinical settings is promising. The optimal cutoff scores for identifying victims and perpetrators were ≥ 5 for total victimization and ≥ 4 for total perpetration subscale scores, respectively. This tool can be applied in school‐based mental health programs, public health research, and adolescent counseling services to screen for bullying‐related behaviors, identify at‐risk individuals, and inform early interventions. It also provides a standardized measure for future studies exploring the prevalence, risk factors, and outcomes of bullying among Bangladeshi adolescents. Addressing the identified limitations through more representative sampling, longitudinal design, rural contextualization, and expansion of validated comparison tools will further solidify the BCS‐A Bangla's role in bullying research and adolescent mental health assessment in Bangladesh.

## Author Contributions

**Humayra Shahjahan**: conceptualization, methodology, data collection, data curation, formal analysis, writing – original draft. **Ahsan Aziz Sarkar**: formal analysis, writing – review and editing. **Shelina Fatema Binte Shahid**: writing – review and editing. **Nahid Mahjabin Morshed**: supervision, project administration. All authors had access to the data and approved the final draft.

## Funding

This study is partially supported by a grant from the Bangladesh Medical University, Dhaka.

## Ethics Statement

The Ethics Committee of Bangladesh Medical University approved this study (Reference—BSMMU/2023/9371). The study adhered to established ethical guidelines throughout.

## Consent

Written informed consent was obtained from all participants' guardians, with assent from adolescents.

## Conflicts of Interest

The authors declare no conflicts of interest.

## Data Availability

The de‐identified dataset and analysis code supporting this study are available on request to the corresponding author.
